# Spatio‐Selective Reconfiguration of Mechanical Metamaterials Through the Use of Dynamic Covalent Chemistries

**DOI:** 10.1002/advs.202407746

**Published:** 2024-10-22

**Authors:** Tansu Abbasoglu, Oliver Skarsetz, Paula Fanlo, Bruno Grignard, Christophe Detrembleur, Andreas Walther, Haritz Sardon

**Affiliations:** ^1^ POLYMAT University of the Basque Country UPV/EHU Joxe Mari Korta Center Avda. Tolosa 72 Donostia‐San Sebastián 20018 Spain; ^2^ Life‐Like Materials and Systems Department of Chemistry Johannes Gutenberg University Mainz Duesbergweg 10–14 55128 Mainz Germany; ^3^ Center for Education and Research on Macromolecules (CERM) CESAM Research Unit Department of Chemistry University of Liège Liège 4000 Belgium; ^4^ FRITCO2T Platform University of Liège Sart‐Tilman B6a Liège 4000 Belgium; ^5^ WEL Research Institute Wavre 1300 Belgium

**Keywords:** dynamic covalent chemistry, elastomers, metamaterials, non‐isocyanate polyurethanes, shape fixing

## Abstract

Mechanical metamaterials achieve unprecedented mechanical properties through their periodically interconnected unit cell structure. However, their geometrical design and resulting mechanical properties are typically fixed during fabrication. Despite efforts to implement covalent adaptable networks (CANs) into metamaterials for permanent shape reconfigurability, emphasis is given to global rather than local shape reconfiguration. Furthermore, the change of effective material properties like Poisson's ratio remains to be explored. In this work, a non‐isocyanate polyurethane elastomeric CAN, which can be thermally reconfigured, is introduced into a metamaterial architecture. Structural reconfiguration allows for the local and global reprogramming of the Poisson's ratio with change of unit cell angle from 60° to 90° for the auxetic and 120° to 90° for the honeycomb metamaterial. The respective Poisson's ratio changes from −1.4 up to −0.4 for the auxetic and from +0.7 to +0.2 for the honeycomb metamaterial. Carbon nanotubes are deposited on the metamaterials to enable global and spatial electrothermal heating for on‐demand reshaping with a heterogeneous Poisson's ratio ranging from −2 to ≈0 for a single auxetic or +0.6 to ≈0 for a single honeycomb metamaterial. Finite element simulations reveal how permanent geometrical reconfiguration results from locally and globally relaxed heated patterns.

## Graphical abstract

**Reprogrammable covalent adaptable network (CAN) metamaterials**: A non‐isocyanate polyurethane‐based CAN elastomer was synthesized to create permanently reconfigurable metamaterials through global as well as photothermal and electrothermal heating. The localized Joule heating results in a heterogeneous distribution of Poisson's ratio within a single metamaterial.

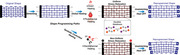

## 1 Introduction

Mechanical metamaterials are artificially engineered materials possessing properties that surpass those found in nature.^[^ ^1^, ^2^ ^]^ These materials exhibit exotic properties such as ultrahigh stiffness at extremely low density,^[^ ^3^ ^]^ negative Poisson's ratios (auxetics),^[^ ^4^, ^5^ ^]^ and negative compressibility,^[^ ^6^, ^7^ ^]^ which are tailored from the rational design of their periodic unit cell architecture rather than composition.^[^ ^8^, ^9^, ^10^ ^]^ The variation in geometrical parameters dictates the sign and magnitude of the Poisson's ratio.^[^ ^5^, ^11^, ^12^, ^13^ ^]^ For instance, honeycomb metamaterials with a unit cell angle θ > 90° exhibit a positive Poisson's ratio under tensile deformation, while their re‐entrant counterparts with θ < 90° show an auxetic response.

Traditionally, the geometry of metamaterials, and consequently their mechanical properties, are determined by the initial design and remain unalterable post‐fabrication. However, recent advancements have led to the development of reconfigurable metamaterials through the integration with responsive materials,^[^ ^14^, ^15^, ^16^, ^17^ ^]^ enabling property changes in response to external stimuli such as heat,^[^ ^18^ ^]^ light,^[^ ^19^ ^]^ or magnetic fields.^[^ ^20^ ^]^

Progress in reconfigurable metamaterials includes the use of shape memory polymers (SMPs), which can fix temporary shapes and recover the original, permanent shape only upon heating.^[^ ^21^, ^22^ ^]^ Metamaterial structures consisting of polymer networks with permanent covalent crosslinks have been deformed above thermal transitions like the glass transition temperature (*T*_g_) and then cooled down while deformed to lock in temporary shapes, although this often results in limited permanent reprogramming due to the entropic elasticity maintaining the network in a stressed state.^[^ ^23^, ^24^, ^25^, ^26^ ^]^ In this case, one cannot expect multiple permanent shape reconfigurations from a single SMP structure. Moreover, high *T*_g_ SMP materials remain glassy at room temperature, preventing extensive stretching.^[^ ^27^ ^]^ Liquid crystal elastomers (LCEs) have been explored for stretchable reconfigurable metamaterials,^[^ ^28^, ^29^ ^]^ kirigami‐inspired devices^[^ ^30^ ^]^ and rolling robots;^[^ ^31^ ^]^ however, they require alignment of mesogens,^[^ ^32^, ^33^ ^]^ and do not support shape‐locking.

To overcome existing limitations, solid‐state plasticity has been leveraged to permanently lock shapes by embedding dynamic covalent bonds within the crosslinked structure, which is commonly referred to as a covalent adaptable network (CAN).^[^ ^34^, ^35^ ^]^ Upon exposure to stimuli, the network rearrangement, characterized by reversible bond formation and breakage, results in irreversible macroscopic deformation. CANs have been recently combined with SMPs to achieve permanent in addition to temporary shape reconfiguration.^[^ ^36^, ^37^, ^38^, ^39^ ^]^ Until now, efforts have primarily focused on reshaping relatively simple and continuous structures with CAN‐based SMP systems, such as twisting a sheet into a spiral or folding it like origami without reprogramming effective mechanical properties.

Dynamic covalent chemistry (DCC) offers a diverse set of synthetic tools, including various reversible bonds, catalysts, and polymer matrices, enabling precise tuning of the timescales and extent of the material's dynamic response.^[^ ^40^ ^]^ The ability to design elastomers with a rapid exchange rate for permanent shape reconfigurability and long‐term dimensional stability—featuring very low creep at lower temperatures—is crucial.^[^ ^40^, ^41^ ^]^ A classic approach to achieving fast exchange kinetics in DCC is the incorporation of intrinsically reactive linking groups, such as aromatic disulfides that can exchange even at room temperature, into the polymer matrix.^[^ ^42^, ^43^ ^]^ It has been demonstrated that aromatic disulfides integrated into a poly(urea‐urethane) (PUU) elastomer are constrained at lower temperatures by hydrogen bonding interactions within the polymer matrix.^[^ ^44^ ^]^ This interplay between dynamic bond exchanges and complementary intermolecular interactions endows the PUU thermoset elastomer with limited creep at room temperature while maintaining excellent reprocessability at elevated temperatures. Notably, auxetic metamaterial elastomers have been developed using a dual material skeleton/matrix architecture, where a rigid PUU skeleton is coupled with a soft PUU matrix through dual interfacial healing to enhance the mechanical properties of auxetic materials.^[^ ^45^ ^]^ Despite these advancements, the permanent shape reconfiguration of elastomeric metamaterials and investigation of the change of effective mechanical properties has not yet been exploited. Furthermore, in applications such as biological implants, a heterogeneously distributed Poisson's ratio is desired and has been achieved by metamaterial design before fabrication.^[^ ^46^, ^47^ ^]^ Dynamic bond exchange reactions that are spatially triggered through localized heating can enable such heterogeneously distributed Poisson's ratios within the metamaterial architecture, which can be gradually adjusted and repeatedly reconfigured after fabrication.

**Scheme 1:**
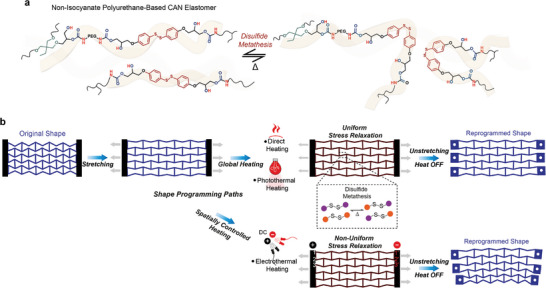
Reprogrammable CAN metamaterials. a) Schematic illustration of the network rearrangement induced by the metathesis of aromatic disulfide bonds. b) Schematic illustration of the shape reconfiguration of the CAN metamaterial for both global and local heating strategies. CAN metamaterial shape programming involves stretching the structure, accompanied by global or local heating. Direct and photothermal heating trigger disulfide metathesis throughout the overall structure, thereby enabling uniform stress relaxation, and, correspondingly, the global permanent shape change. The structure can also be reprogrammed locally. Electrothermal heating, where the electrodes are selectively positioned at either end of the structure (lines 1–3), enables spatially controlled heating and thus non‐uniform stress relaxation.

In this study, we introduce elastomeric CAN systems to metamaterial architectures to achieve reprogrammability of the metamaterial architecture and the resulting Poisson's ratio. The novelty of our work lies in the following aspects: i) This is the first work to use spatio‐selective electrothermal heating to reconfigure a CAN metamaterial specimen after fabrication to achieve a heterogeneous distribution of Poisson's ratio. Although CANs have been explored in additive manufacturing of various lattice structures, global and local reprogramming with resulting changes in effective mechanical properties remains elusive.^[^ ^48^, ^49^, ^50^ ^]^ ii) Finite element (FE) simulation further elucidates the distribution of stress during deformation as well as the global and local stress relaxation. iii) The elastomeric CAN system is based on non‐isocyanate polyurethane (**Scheme** **1**a) and derived from sustainable precursors. We have chosen poly(hydroxy urethane) (PHU) as our polymer matrix due to its customizable, CO_2_‐sourced precursors^[^ ^51^ ^]^ and its capacity to form a hydrogen‐bonded network through its multiple pendant hydroxyl groups.^[^ ^52^ ^]^ By incorporating a disulfide monomer, we exploit aromatic disulfide metathesis for rapid stress relaxation. We achieve spatial control over deformation patterns using CNTs deposited on the metamaterials.

Our system allows for the permanent reconfiguration of honeycomb and re‐entrant auxetic structures through environmental temperature increases and photothermal and electrothermal triggers (Scheme 1b). The reprogramming process involves uniaxial stretching followed by heating, which activates disulfide metathesis across the entire structure for uniform stress relaxation and permanent shape retention. Additionally, localizing electrothermal heating with deliberate electrode placement allows for gradient shape formation from a uniformly deformed state, resulting in a gradational variation of the Poisson's ratio within a single metamaterial system.

## 2 Results and Discussion

### 2.1 Reconfigurable Dynamic Covalent Elastomer Design

**Figure 1:**
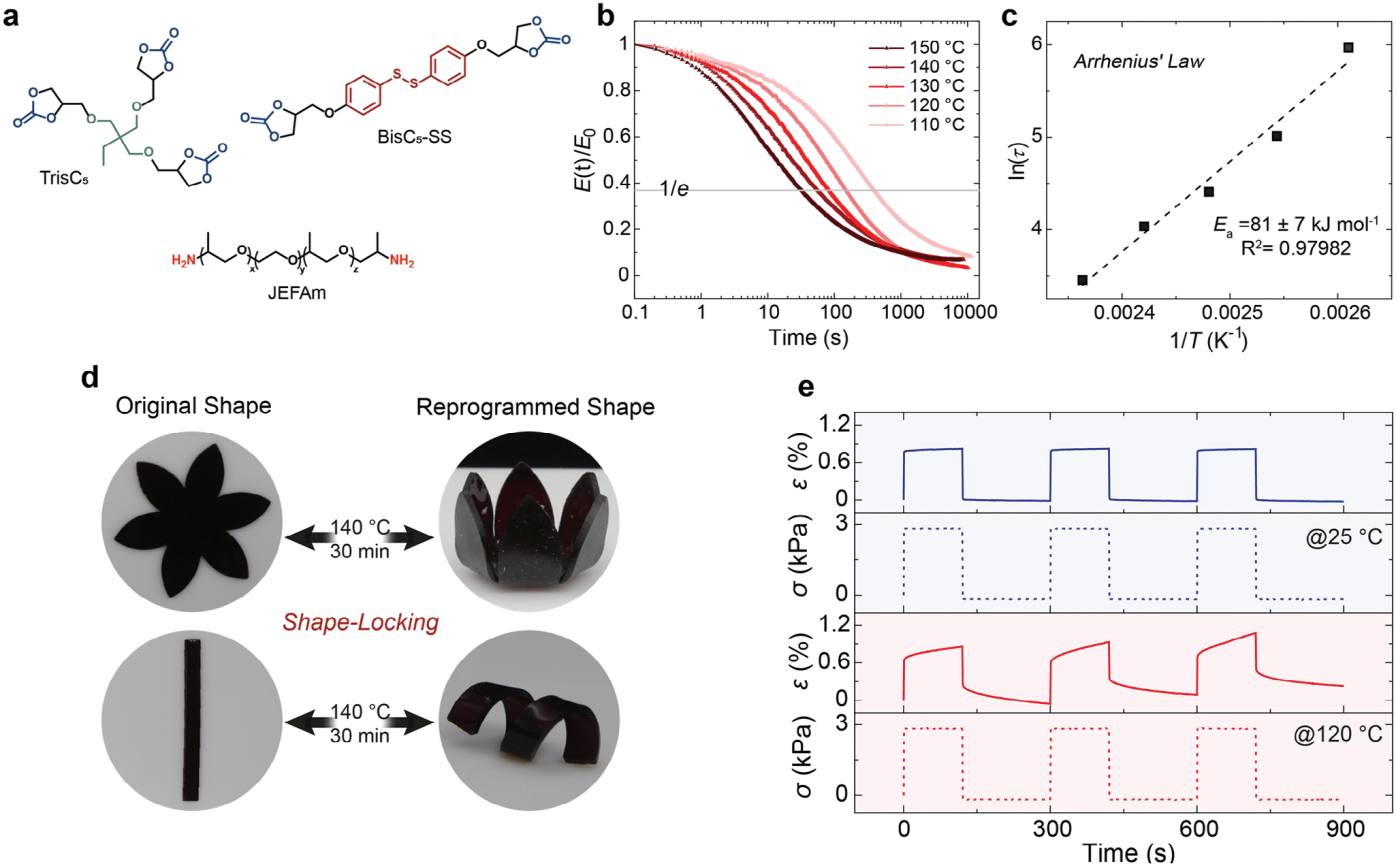
Precursors' structure and characterization of the aromatic disulfide containing PHU. a) Chemical structure of the monomers used to synthesize the PHU elastomer. b) Normalized stress relaxation curves of PHU at 4% strain and different temperatures. The relaxation times are determined at *E*/*E*_0_= 1/e, marked by a light gray line. c) Fitting the relaxation times τ to the Arrhenius equation (Equation S1, Supporting Information). From the fitted curves, we calculated the activation energy (*E_a_* = 81 ± 7 kJ mol^−1^) for the exchange reaction. d) Left, lotus flower and rectangular strip‐shaped samples; right, plasticity‐based permanent shape reconfigurability at elevated temperature (140 °C for 30 min). e) Cyclic creep recovery profiles of the elastomer at 25 °C and 120 °C at varying the stress between 0 and 2.8 kPa (dashed line: stress; solid line: strain).

To produce an elastomeric CAN material, we synthesized a PHU elastomer through polyaddition of five‐membered polycyclic carbonates (5‐CC) with soft elastomeric propylene oxide‐capped poly(ethylene glycol)‐based diamine, JEFAm (Jeffamine ED‐900),^[^ ^53^ ^]^ in the presence of 1,8‐diazabicyclo[5.4.0]undec‐7‐ene (DBU) as shown in **Figure** **1**a. Tris(cyclic carbonate) (trimethylolpropane triscarbonate), referred to as TrisC_5_, was used as a crosslinker. We also added a dicyclic carbonate‐bearing aromatic disulfide bond (BisC_5_‐SS) to enhance relaxation time and enable self‐healing. CO_2_‐derived 5‐CCs, emerging as sustainable building blocks for elastomeric urethane‐based CAN, were prepared by coupling CO_2_ with the corresponding epoxides in quantitative yield under solvent‐free conditions. ^1^H and ^13^C NMR spectroscopy confirmed their chemical structures (see the Experimental Section, Figures S3–S5, Supporting Information).

Attenuated total reflection infrared (ATR‐IR) spectroscopy confirmed the formation of the network, as detailed in Figure S6 (Supporting Information). The crosslinked PHU CAN exhibited a nearly complete disappearance of the carbonate bands at ≈1800 cm^−1^ of the 5‐CC precursors, with a new band appearing at 1715 cm^−1^ corresponding to the C═O urethane stretch. Dynamic mechanical analysis (DMA) demonstrated the elastomeric nature of the material, with a glass transition temperature *T*_g_ of −33 °C, as shown in Figure S7 (Supporting Information). Next, the mechanical performance of the PHU elastomer was explored by a sequential cyclic tensile test with increasing strains (Figure S8a, Supporting Information). The residual deformation after strains up to 90% remains below 6%, and negligible hysteresis or degradation in modulus after each cycle (Figure S8b, Supporting Information), showing that the synthesized PHU demonstrates good elastic performance.

Reprogramming of a PHU elastomer containing only TrisC_5_ crosslinker and JEFAm is theoretically possible; however, the polyurethane transcarbamoylation is too slow and results in incomplete stress relaxation (Figure S9 in the Supporting Information). Incorporating aromatic disulfide bonds through the BisC_5_‐SS monomer dramatically increased the rate of network stress relaxation. Stress relaxation tests at elevated temperatures revealed relaxation times τ decreasing from 398 s at 110 °C to 32 s at 150 °C, according to the Maxwell model (Figure 1b). An activation energy of 81 kJ/mol^−1^ ± 7 kJ/mol^−1^, which reflects the energy for disulfide metathesis,^[^ ^54^ ^]^ was determined using Arrhenius' law (Figure 1c). To demonstrate permanent shape reprogramming, we shaped the PHU elastomer into a flower and a linear strip by mold‐casting the dissolved monomer, crosslinker, and catalyst into PTFE molds and cured at 80 °C for 72 h (Figure 1d). Subsequently, we deformed the initially flat flower into a closed shape and twisted the linear strip. We then heated both structures at 140 °C for 30 min to activate the dynamic covalent bond exchange, resulting in macroscopic stress relaxation and thus permanent reprogramming. Cyclic creep recovery experiments provided further insight into temperature‐dependent elastoplastic deformation. For these experiments, we applied cyclic stresses of σ = 2.8 kPa to the CAN at both 25 °C and 120 °C, while measuring the resulting strain (Figure 1e). Similar to rubber, the PHU CAN completely recover to 0% strain in repeated stress cycles at 25 °C.^[^ ^55^ ^]^ However, due to heat‐triggered dynamic disulfide metathesis at 120 °C, the PHU CAN permanently deform with each stress cycle, accumulating a plastic deformation of 0.2% after three cycles. To demonstrate the self‐healing capability, a dogbone sample was first cut in the middle, and then the edges of the separated parts were brought into contact, followed by heating at 140 °C for 30 min (Figure S10a, Supporting Information). As illustrated in Figure S10b (Supporting Information), the healed sample could retain 126% strain with a healing efficiency η of 81%.

### 2.2 Exploiting DCC for Shape Fixing and Self‐Healing

**Figure 2:**
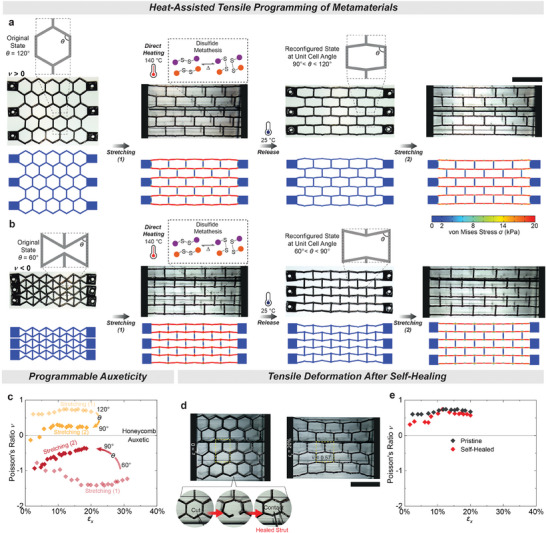
Permanent shape reconfiguration and self‐healing of the dynamic PHU metamaterials. a, b) Experiments and FE simulation of the reprogramming process. As a first step, the honeycomb (θ = 120°) and auxetic re‐entrant (θ = 60°) architectures in the fully stretched state undergo reprogramming at 140 °C for 20 min, wherein dynamic disulfide metathesis proceeds. Once stress is removed, these CAN structures retain their predefined shapes with an angle of the unit cells near θ = 90°. Finally, the new permanent shapes are subjected to a second round of stretching at room temperature. The scale bar is 30 mm. FE reveals the local stress during deformation, which is released during stress relaxation. c) Tunable auxetic behavior of the CAN metamaterials, where the extent of auxeticity depends on the unit cell angle θ. The Poisson's ratios of both metamaterials approach zero as their unit cell angles are reprogrammed into θ = 90°. d, e) An individual strut cut with a blade into two sections and self‐healed after reconnecting two parts of the cut strut at 140 °C for 30 min to restore the geometrical integrity of the honeycomb metamaterial. The pristine and self‐healed geometries show comparable values of Poisson's ratio (0.60 ± 0.07 at ε_x_ = 20%). The scale bar is 30 mm. For the experiment described above, the Poisson's ratio ν is calculated by ν = − ε_y_/ε_x_, where x is the axial (loading) direction and y is the lateral direction (see the calculation details in the Supporting Information).

After validating the stress relaxation and permanent reprogramming of the PHU CAN networks, regular and re‐entrant honeycomb metamaterials were fabricated by casting in PTFE molds. For reprogramming, the metamaterial structures were stretched at room temperature, followed by heating (Figure 1a,b). Heating to 140 °C permanently fixed the deformed shapes by inducing network rearrangement and stress relaxation in the polymer network through aromatic disulfide metathesis. As a result of the reprogramming, the honeycomb metamaterial's initial unit cell angle θ decreased from 120° to ≈90° under applied tensile deformation (**Figure** **2**a). We conducted finite element (FE) simulations to gain further insight into the stress distribution under tensile deformation and subsequent stress relaxation during direct heating (Figure 2a). Experimentally determined material parameters such as activation energy, resistivity, and stiffness were used as input parameters, as listed in Table S1 (Supporting Information). During stretching, the stress increases mainly along the horizontal lines. Direct heating relaxes the stress and permanently fixes the shape (Figure 2a). The character of the metamaterial and its reprogramming becomes evident when comparing the Poisson's ratios during deformation, and before and after reprogramming. Before reprogramming, the Poisson's ratio ν remained constant at ≈0.7 with increasing ε_x_ strain up to 20% (Stretching (1); Figure 2c). When maintaining the strain at this level and heating the material at 140 °C for 20 min, the new shape was permanently set. Since the stress fully relaxed, the newly reconfigured metamaterial, now with θ = 90°, could subsequently be deformed to higher strain values. The resulting Poisson's ratio reached a lower value of 0.24 with increasing ε_x_ strain up to 20% (Stretching (2); Figure 2c). The Poisson's ratio at the end of the first stretch is higher than at the beginning of the second stretch due to the difference in stress before and after stress relaxation. The small deviation in Poisson's ratio at low strain stems from clamping the metamaterial in a slightly buckled state. Similarly, the auxetic re‐entrant metamaterial exhibited comparable behavior (Figure 2b). The original metamaterial, with θ = 60°, reached negative Poisson's ratio values of up to ν = −1.4 with increasing ε_x_ strain up to 20% (Stretching (1); Figure 2c). After deformation, this metamaterial was also heated at 140 °C for 20 min to permanently lock it in its reconfigured unit cell angle of θ = 90°. The reconfigured metamaterial then showed increased Poisson's ratio values of up to ν = −0.4 (Stretching (2); Figure 2c). Like the honeycomb metamaterial, the Poisson's ratio at the end of the first stretch slightly deviates from the beginning of the second stretch due to the differences in stress before and after stress relaxation.

Next, we capitalize on the self‐healing capacity of the CAN to repair a damaged metamaterial strut using the same dynamic aromatic disulfide metathesis chemistry (Figure 2d). Initially, the pristine metamaterial exhibited a Poisson's ratio of approximately ν = 0.6 when deformed to ε_x_ = 20% (Figure 2e). To demonstrate self‐healing, one strut was cut in half with a razor blade after the deformation was removed (Figure 2d). The separated parts were manually pressed together for a few seconds and then placed in an oven without any further fixation at 140 °C for 30 min. Once healed, stretching the metamaterial to ε_x_ = 20% resulted in a similar Poisson's ratio of around ν = 0.6 (Figure 2e). Minor deviations of Poisson's ratio at small strain stem from minor differences in the clamping of the sample.

### 2.3 Global and Local Heating Strategies for Reprogramming

**Figure 3:**
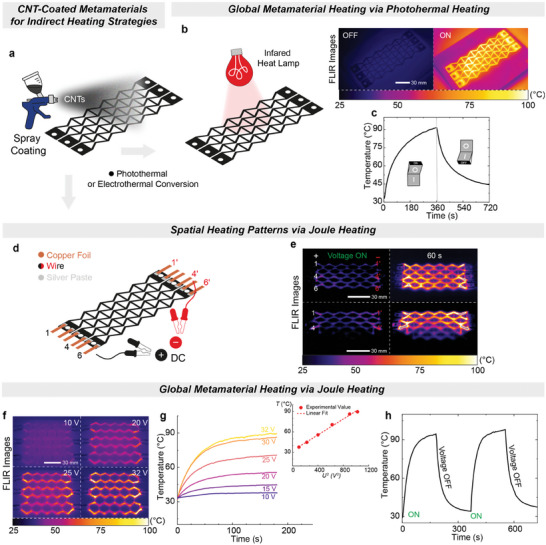
Global and local heating approaches via electrothermal/photothermal effects of the CNTs‐coated PHU composite metamaterials. a) Schematic preparation of the PHU metamaterials coated with a layer of CNTs by a simple spray‐coating approach for indirect heating through photothermal effect or Joule heating. b, c) Photothermal conversion performance of the auxetic composite placed under IR light illumination. d) Circuit schematic of the experimental setup with copper electrodes glued onto both ends for Joule heating. Silver paste (grey circles) is applied between the copper foil and the metamaterial. e) Thermal imaging photographs of the coated auxetic structure showing the spatially controlled increase of the temperature with different electrode placements. It is important to note that the current heats the metamaterial, with the heating effect being more pronounced in its horizontal lines. f) Joule heating‐based thermal map series captured at 10 V, 20 V, 25 V, and 32 V DC voltages using an IR camera. g) Time‐dependent temperature profiles at various applied voltages. Inset: linear fitting profile between the steady‐state temperatures and the square of the voltages (using the following equation: *T_s_* = *T*_0_ + *U* ^2^/*R*α*A*). h) Heating/cooling cycles under an applied voltage of 32 V.

After successfully reprogramming the metamaterial using global heating, we further demonstrated the application of photothermal and electrothermal heating for precise, on‐demand spatial reprogramming of the metamaterial's geometry and mechanical response.^[^ ^56^, ^57^, ^58^ ^]^ To achieve this, single‐wall carbon nanotubes (CNTs) were deposited onto the metamaterial by spray coating, forming a photo‐ and electro‐active layer (**Figure** **3**a). The CNT layer is thin in comparison to the 2 mm thick elastomeric metamaterial and thus does not influence the deformation pattern. This coating enabled the disulfide metathesis reaction upon heating, which relaxed the applied stress and thus locked the macroscopic shape of the metamaterial based on local photothermal or electrothermal activation. The temperature distribution within the composite metamaterials was recorded using a forward‐looking infrared (FLIR) camera. The CNT‐coated metamaterial generated a global heating pattern during photothermal heating, equivalent to direct oven heating (Figure 3b). Upon exposure to infrared light (IR) with a wavelength greater than 700 nm and an intensity of 6 W cm^−2^, the metamaterial's temperature rose to 92 °C within 180 s (Figure 3c), rapidly dissipating within a few minutes after the light was turned off. To achieve inhomogeneous heating patterns, CNTs could, in principle, be deposited with spatial control onto the metamaterials.

Electrothermal heating provided the advantage of spatially reprogrammable heating patterns by altering localized electrical stimulation across the global CNT coating. Initially, we attached three positive (+) and negative (−) electrodes to the two ends of the CNT‐coated metamaterial, specifically at locations 1, 4, and 6 and 1′, 4′, and 6′, corresponding to six individual horizontal lines (Figure 3d). The application of a 32 V direct current (DC) power source resulted in the global specimen heating to 69 °C within 60 s (Figure 3e, top panel). Upon removing the electrodes positioned at 6 and 6′, we observed spatial differences in the heating patterns across the material. Consequently, Line 1 heated to 96 °C, while Line 6 remained at 25 °C (Figure 3e, bottom panel).

To quantitatively understand the Joule heating effect in the CAN metamaterials, we monitored the average temperature across the horizontal lines at different input voltages using a 3 by 3 electrode configuration (Figure 3f). The time‐temperature curves showed an increase in temperature with higher applied voltages (Figure 3g). Specifically, the temperature reached 90 °C at 32 V, while an input voltage of 10 V limited the temperature to 40 °C. The linear dependence of the steady‐state temperature on the squared voltage *U* ^2^ demonstrated the direct relationship between electrothermal conversion and the applied voltage (inset in Figure 3g; Equation S2, Supporting Information). Furthermore, temperature control was achieved temporally through repeated ON‐OFF cycles, with temperatures reaching up to 96 °C ± 2 °C using a 32 V input voltage (Figure 3h).

### 2.4 Electrothermal Local Shape Reprogramming

**Figure 4:**
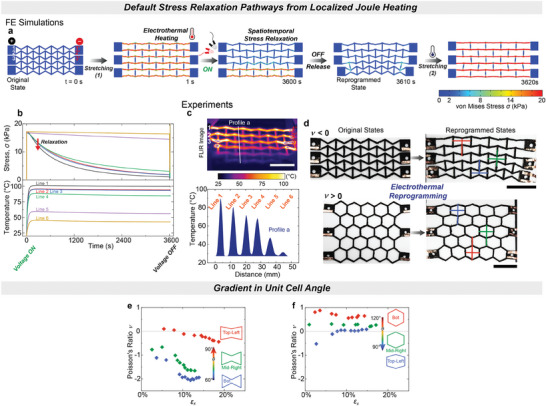
Programmable local Joule heating phenomenon enables spatially varying structural properties. a) FE simulations for the stress distribution of the auxetic CNTs‐coated PHU architecture during and after shape reconfiguration into a lock‐in state. The electric connections are between the labeled positions 1–1′, 2–2′, and 3–3′ (t = 0 s). With the applied voltage of 32 V for 3600 s, the re‐entrant auxetic structure under stretching shows the local stress relaxation, corresponding to the placement of the electrodes. Subsequently, the electricity power supply is turned off, and the tensile load is removed. The resultant structure is heterogeneously reprogrammed and re‐stretched at room temperature (3620 s). b) Temperature evolution and the trends of the stress relaxation in each line during the simulated Joule heating. Stress level throughout the reconfigurable architecture increases abruptly upon re‐stretching. The tensile stress vanishes (red arrow) in the Joule‐heated lines up to 85 °C and higher, including lines 1–4. c) Spatial temperature distribution of the auxetic composite along the marked white line (profile a), extracted from the thermal image of the auxetic CNTs‐coated PHU elastomer under stretching. The scale bar is 30 mm. d) Experimental results from the electrothermal reprogramming of the CAN architectures, match the FE simulation results. The cross XY configurations represent the top, middle, and bottom lines. e, f) Spatially varying Poisson's ratios along the reshaped metamaterial architectures with respect to the unit cell angle θ. The local Pisson's ratios are calculated from the numerical values on top‐left (red), mid‐right (green), and bot (blue) unit cells during the re‐stretching.

Next, we conducted FE simulations to gain further insight into the stress distribution under tensile deformation and subsequent stress relaxation during local electrothermal heating (**Figure** **4**a,b). The horizontal lines of the metamaterial corresponded to the locations of highest stress, while its vertical struts did not carry any stress at 1 s and 3620 s; as the metamaterial was stretched horizontally (Figure 4a). This alignment coincided with the path of the current and, consequently, the Joule heating (Figure S11, Supporting Information), facilitating the horizontal relaxation of stress at 3600 s (Figure 4a). Locally, the temperature rose up to 100 °C in Line 1, triggering rapid stress relaxation (Figure 4b). In Line 6, the temperature remained ≈40 °C, resulting in slower stress relaxation (Figure 4b). Only lines 1–4 reached the critical temperature of 60 °C, which was sufficient to trigger almost complete stress relaxation within 1 h (Figure 4b; Figure S10a,b, Supporting Information). After releasing the tensile deformation in the FE simulation, inhomogeneous shape reprogramming was observed at 3610 s (see Figure 4a; Figure S12a, Supporting Information).

In the experiments, the metamaterial specimens were deformed to the desired extension, followed by the application of a DC voltage while in the deformed state. The re‐entrant auxetic metamaterial, with electrodes connected between lines 1, 2, and 3, exhibited localized peak temperatures in the cross‐sectional steady‐state temperature profile along the vertical axis (Figure 4c). The peak temperatures along Line 1 and Line 2 reached up to 90 °C, while those along Lines 3 and 4 reached 72 °C. The temperature along Line 6 remained close to room temperature. This temperature gradient along the vertical axis further defined the stress relaxation. After 1 h of Joule heating, the applied strain was released through the disulfide metathesis reaction. Similar to the FE simulations, this process achieved spatial reprogramming, as shown in Figure 4d. Upon removal of the applied stress, the initially homogeneous unit cell angles of 60° varied along the vertical axis (Figure 4d).

In the metamaterial, lines experiencing the highest temperatures solidified their deformed configuration, resulting in a unit cell angle of 75° ± 2°. Similarly, a comparable temperature gradient was observed in the honeycomb structure (Figure S12b,c, Supporting Information). As observed previously, the lines at higher temperatures maintained their reprogrammed deformed state, while the lines at lower temperatures recovered elastically (Figure 4d). After the reprogramming process, the angle of both the re‐entrant auxetic and honeycomb metamaterials remained nearly unchanged in the bottom unit cells, whereas the angles in the top and middle unit cells approached θ = 90° (Figure 4d).

Crucially, the locally reprogrammed metamaterial unit cell angles led to local variations in Poisson's ratio when the metamaterials were stretched a second time. The gradient in unit cell angles along the vertical axis resulted in a corresponding gradient in Poisson's ratio values (Figure 4e,f). The Poisson's ratio of the auxetic metamaterial under applied deformation varied from ν = −2 to nearly zero within the same specimen (Figure 4e). In the honeycomb metamaterial, the Poisson's ratio ranged from ν = 0.6 to zero, also within the same specimen (Figure 4f).

## 3 Conclusion

In summary, our study demonstrates the significant potential of dynamic covalent chemistry in the reprogramming of metamaterial geometries, mechanical responses, and self‐healing through targeted thermal interventions. We showed that reconfiguration of the angle of the re‐entrant auxetic and honeycomb unit cell structures by heating stretched metamaterials reprograms their respective negative and positive Poisson's ratios to near‐zero values at θ = 90°.

Additionally, we explored the use of a deposited CNT layer to enable both photothermal and electrothermal heating methods. This allowed us to create varied heating patterns through localized Joule heating by adjusting electrode placements, leading to spatially different stress relaxation and thus heterogeneous auxetic properties within a single structure. FE analysis provided essential guidance for our experimental design and offered insights into the local stress dynamics during electrothermal stress relaxation.

The ability to control stimuli at the unit cell level introduces a new degree of control within a single metamaterial architecture. Looking forward, the development of more advanced dynamic covalent chemistries, potentially responsive to different stimuli, could enhance the adaptability of active metamaterials. Metamaterials with heterogenic auxetic properties hold promise in shape‐matching and form‐fitting materials and applications like energy‐dissipating structures, conformal electronic patches, and soft robotics. Our findings suggest a path toward smarter and more adaptable materials.

## 4 Experimental Section

All methods are described in the Supporting Information.

## Conflict of Interest

The authors declare no conflict of interest.

## Author Contributions

T.A. and O.S. contributed equally to this work. O.S. and A.W. conceptualized the project. T.A., O.S., and A.W. designed the experiments. T.A. developed and synthesized the polymer networks. T.A. and O.S. developed the metamaterial fabrication and different heating techniques. P.F. synthesized the disulfide epoxy monomer precursor. B.G. and C.D. synthesized the cyclic carbonates. T.A. and O.S. analyzed data and interpreted results. O.S. conducted finite element simulations. T.A. wrote the manuscript, which was revised and edited by O.S., H.S., and A.W. All authors commented on the manuscript.

## Supporting information



Supporting Information

## Data Availability

The data that support the findings of this study are available from the corresponding author upon reasonable request.
