# Supplementary material for: Spatio‐Selective Reconfiguration of Mechanical Metamaterials Through the Use of Dynamic Covalent Chemistries
Source: Adv Sci (Weinh). 2024 Oct 22;11(45):2407746. doi: 10.1002/advs.202407746 (PMC11615789; doi:10.1002/advs.202407746)
Supplement: Supplementary file 1 — Supporting Information [file ADVS-11-2407746-s001.docx]

**Supporting Information**

**Spatio-Selective Reconfiguration of Mechanical Metamaterials Through Facile Aromatic Disulfide Metathesis**

Tansu Abbasoglu^+1^, Oliver Skarsetz^+2^, Paula Fanlo^1^, Bruno Grignard^3,4^, Christophe Detrembleur^3,5^, Andreas Walther*^2^, Haritz Sardon*^1^

^+^These authors contributed equally to this work.

^1^T. Abbasoglu, Paula Fanlo, H. Sardon

POLYMAT, University of the Basque Country UPV/EHU, Joxe Mari Korta Center, Avda. Tolosa 72, 20018 Donostia – San Sebastián, Spain

E-mail: [haritz.sardon@ehu.eus](mailto:haritz.sardon@ehu.eus)

^2^O. Skarsetz, A. Walther

Life-Like Materials and Systems, Department of Chemistry, Johannes Gutenberg University Mainz, Duesbergweg 10–14, 55128 Mainz, Germany

E-Mail: [andreas.walther@uni-mainz.de](mailto:andreas.walther@uni-mainz.de)

^3^B. Grignard, Christophe Detrembleur

Center for Education and Research on Macromolecules (CERM), CESAM Research Unit, Department of Chemistry, University of Liège, 4000 Liège, Belgium

^4^FRITCO_2_T Platform, University of Liège, Sart-Tilman B6a, 4000 Liège, Belgium

^5^WEL Research Institute, Wavre 1300, Belgium

**1 Experimental Section**

*Materials:* Epichlorohydrin (ECH), benzyltriethylammonium chloride (TEBAC, 99 %), tetrabutylammonium iodide (*n*Bu_4_NI, ≥ 99 %), sodium hydroxide (NaOH, ≥ 97%), magnesium sulfate anhydrous (MgSO_4_, ≥ 99.5 %) 1,8-Diazabicyclo[5.4.0]undec-7-ene (DBU, 98 %) were purchased from Sigma Aldrich. JEFFAMINE ED-900 (JEFAm) was used as received from Sigma Aldrich. Carbon dioxide (CO_2_) N45 was supplied by Air Liquid. Bis(4-hydroxyphenyl)disulfide (BHPDS) was purchased from BLD Pharmatech GmbH. Single-walled nanotubes (CNTs) with carboxyl functionalization (1 – 2 nm outside diameter, 3 wt% COOH groups) was purchased from SkySpring Nanomaterials. All chemicals were used as received without any further purification.

*Synthesis of bis(4-glycidyloxyphenyl)disulfide (Cyclic Carbonate* ***Precursor****):*


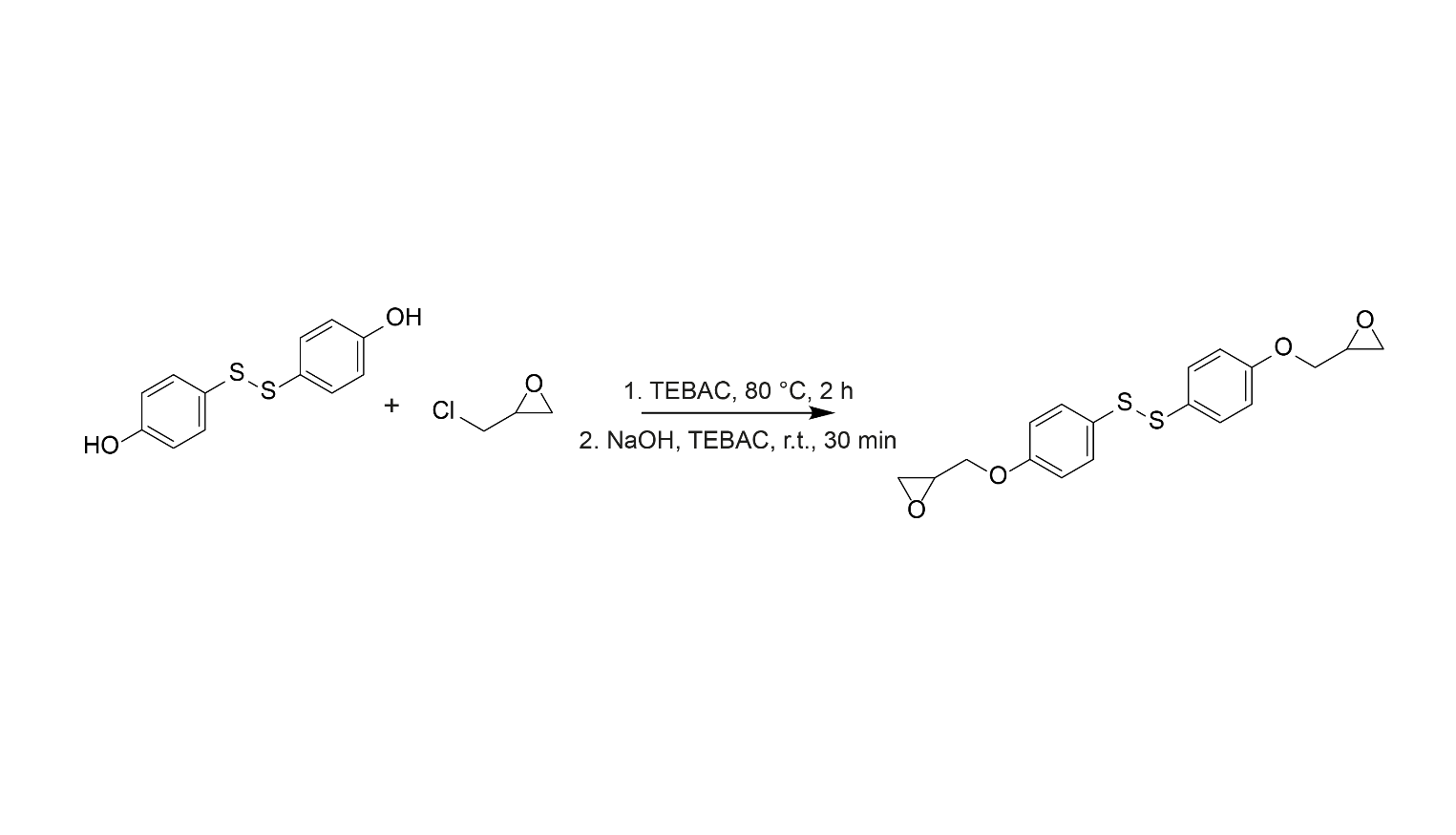


**Scheme S1.** Synthetic route for the cyclic carbonate precursor.

BHPDS (1 equiv.) is dissolved in ECH (10 equiv.) and mixes with TEBAC (0.1 equiv.). The solution is stirred at 80°C for 2 hours. After cooling to room temperature, 5 M NaOH aqueous solution (2.7 equiv.) and TEBAC (0.1 equiv.) are added to the reaction mixture and stirred for 30 min. Thereafter, the crude mixture is extracted with EtOAc. Drying of the organic layer over MgSO_4_ and concentration *in vacuo* afford the diepoxy monomer **precursor** (pale-yellow solid, yield = 90 %). **^1^H NMR** (300 MHz, DMSO-*d_6_*): *δ* 7.40 (m, 4H, Ar-*H*), 6.99 (m, 4H, Ar-*H*), 4.30 (dd, 2H, Ar-OCH_2_), 3.84 (dd, 2H, Ar-OC*H_2_*), 3.31 (m, 2H, OC*H*, oxirane), 2.83 (dd, 2H, OC*H_2_*, oxirane), 2.70 (dd, 2H, OC*H_2_*, oxirane). **^13^C NMR** (300 MHz, DMSO-*d*_6_): *δ* 158.60 (Ar-*C*O), 131.85 (Ar-*C*), 127.36 (Ar-*C*-S), 115.54 (Ar-*C*), 69.10 (O*C*H_2_), 49.55 (O*C*H, oxirane), 43.72 (O*C*H_2_, oxirane).

*Synthesis of the Cyclic Carbonate Monomers:*

4,4'-(((disulfanediylbis(4,1-phenylene))bis(oxy))bis(methylene))bis(1,3-dioxolan-2-one) (**BisC_5_-SS**)


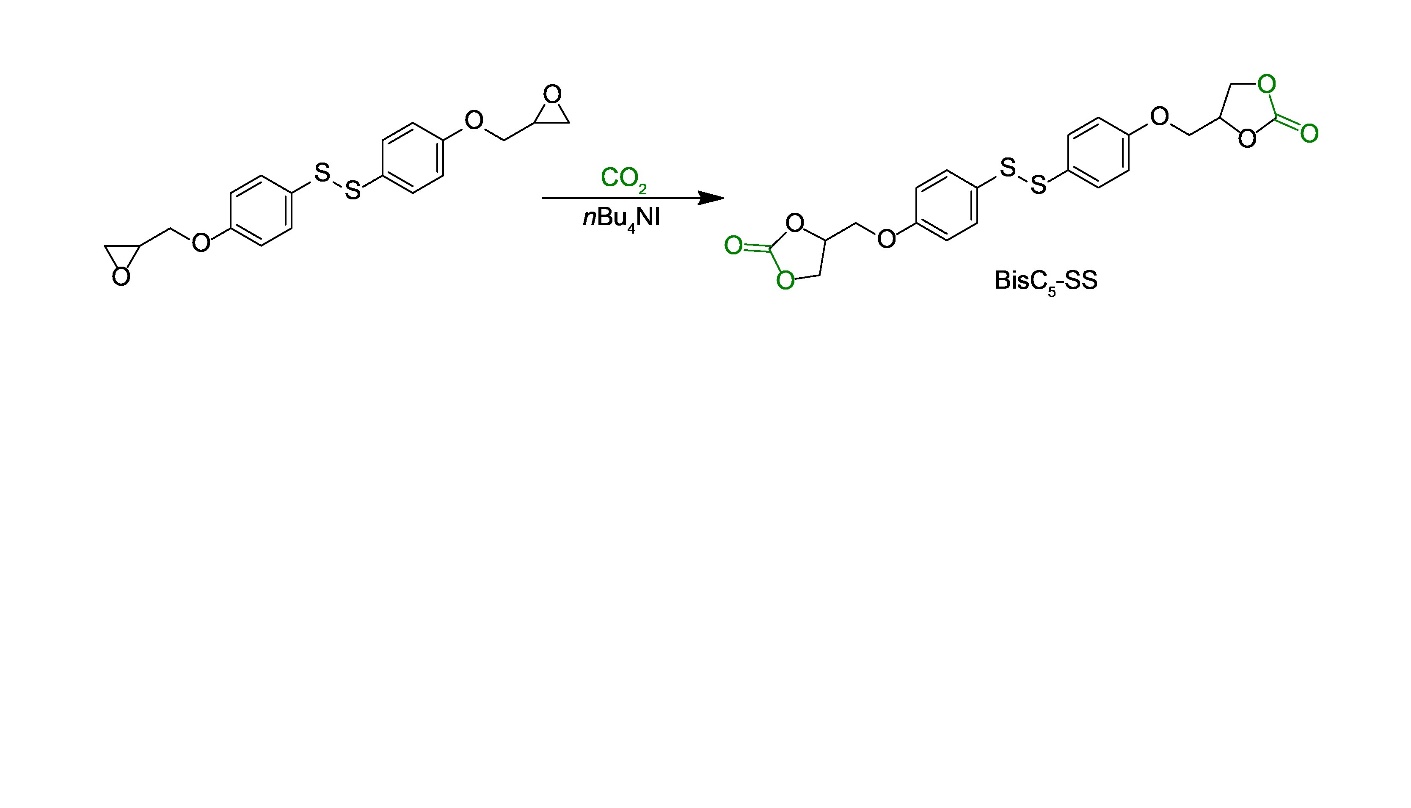


**Scheme S2.** Coupling of bis(4-glycidyloxyphenyl)disulfide (**1**) with CO_2_ to deliver BisC_5_-SS.

A 40 mL reactor equipped with a magnetic stirrer is charged with 1 g of the synthesized cyclic carbonate **precursor** and then 0.0509 g *n*Bu4NI (5 mol%). The reactor is sealed, and the mixture is heated at 110 °C under a constant CO_2_ pressure of 50 bar while stirring. After 24 h, the reactor is carefully depressurized, and the product **BisC_5_-SS** is collected as a yellow solid. The ^1^H NMR characterization (in DMSO-*d_6_*) highlights the total transformation. The product is used without any purification. **^1^H NMR** (300 MHz, DMSO-*d*_6_): *δ* 7.42 (m, 4H, Ar-*H*), 7.01 (m, 4H, Ar-*H*), 5.15 (p, 2H, (C=O)OC*H*), 4.63 (dd, 2H, (C=O)OC*H_2_* ), 4.39 (dd, 2H, (C=O)OC*H_2_*), 4.29 and 4.25 (m, 4H, Ar-OC*H_2_*).


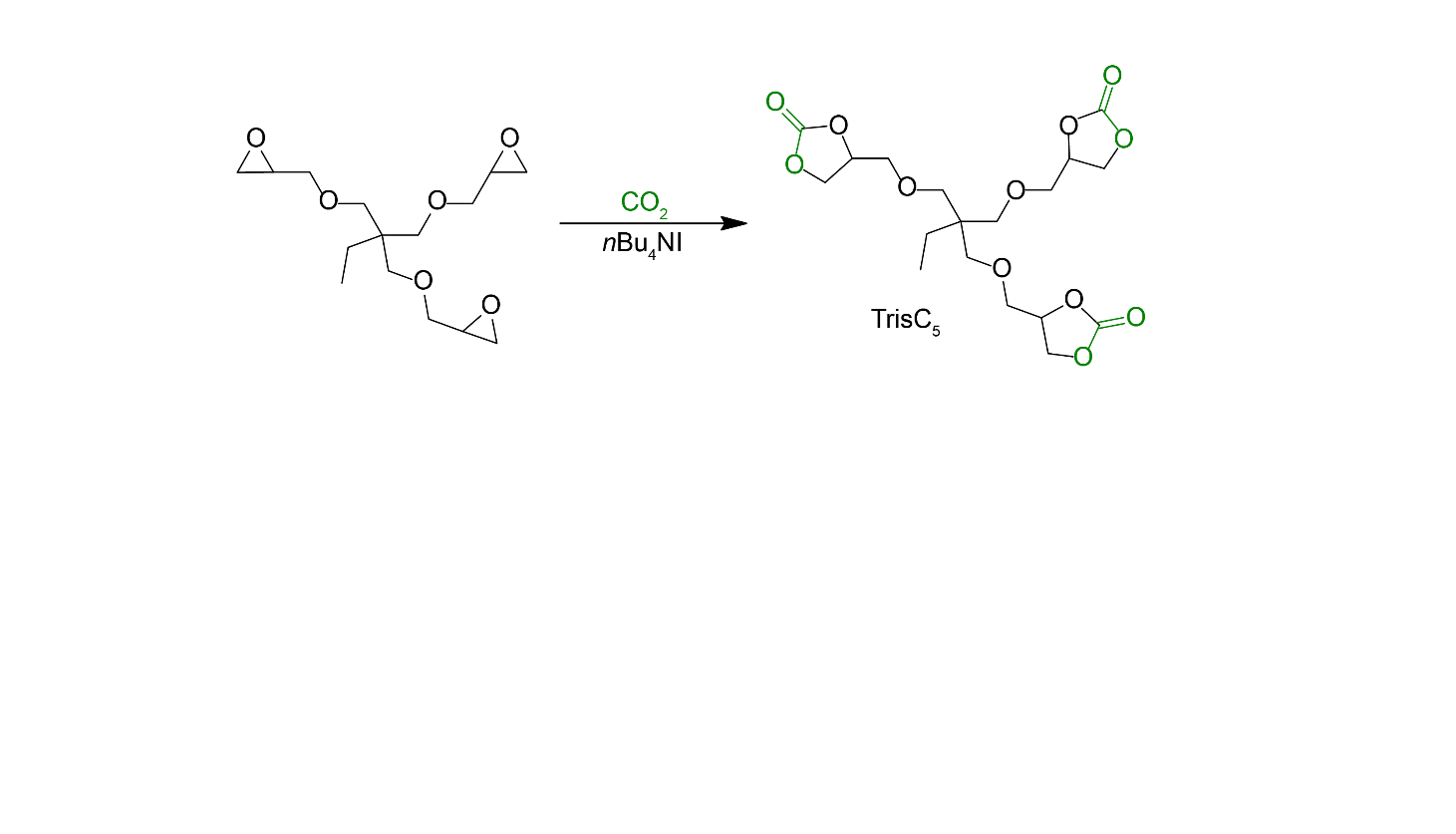


**Scheme S3.** Coupling of trimethylolpropane triglycidyl ether with CO_2_ to deliver TrisC_5_.

4,4’-(((2-ethyl-2-(((2-oxo-1,3-dioxolan-4-yl)methoxy)methyl)propane-1,3diyl)bis(oxy))bis(methylene))bis(1,3-dioxolan-2-one) (**TrisC_5_**) is synthesized by coupling CO_2_ to trimethylolpropane triglycidyl ether following a reported procedure)^[1]^  After collection, **TrisC_5_** is degassed overnight under vacuum and then used without further purification. As previously reported, the resonances between 4.2 ppm and 5 ppm of equivalent intensity corresponds to the cyclic carbonate.

*Synthesis of the Dynamic Covalent PHU Elastomer or Reconfigurable Elastomer Architecture:* The BisC_5_-SS in powder form is dissolved in anhydrous DMSO at a molar concentration of 10 mol L^-1^ using a vortex (VWR standard mini Vortex). The BisC_6_-SS solution, TrisC_5_ and JEFAm are then mixed at 80 °C under constant stirring until the mixture is homogeneous. The molar ratio of TrisC_5_/BisC_5_-SS/JEFAm was set at 1/0.35/1.85. After adding DBU (5 mol% compared to cyclic carbonates), the reaction mixture is carefully filled into the custom-made PTFE mold using an adjustable micropipette and oven-cured at 80 °C for 72 h. The relaxation times were fitted to the Arrhenius equation:

$\tau= \tau_{0}exp(\frac{E_{a}}{RT})$ (S1)

*Manufacturing of PTFE Molds:* The re-entrant auxetic and regular (non-re-entrant) honeycomb geometries are designed in Autodesk Inventor 2024 and milled into PTFE blocks (X × Y × Z =120 × 100 × 10 mm^3^) using a computer numerical control (CNC) machine with a 1 mm diameter milling tool. Dimensions for both geometries with a depth of 2.5 mm are depicted in Figure S1.


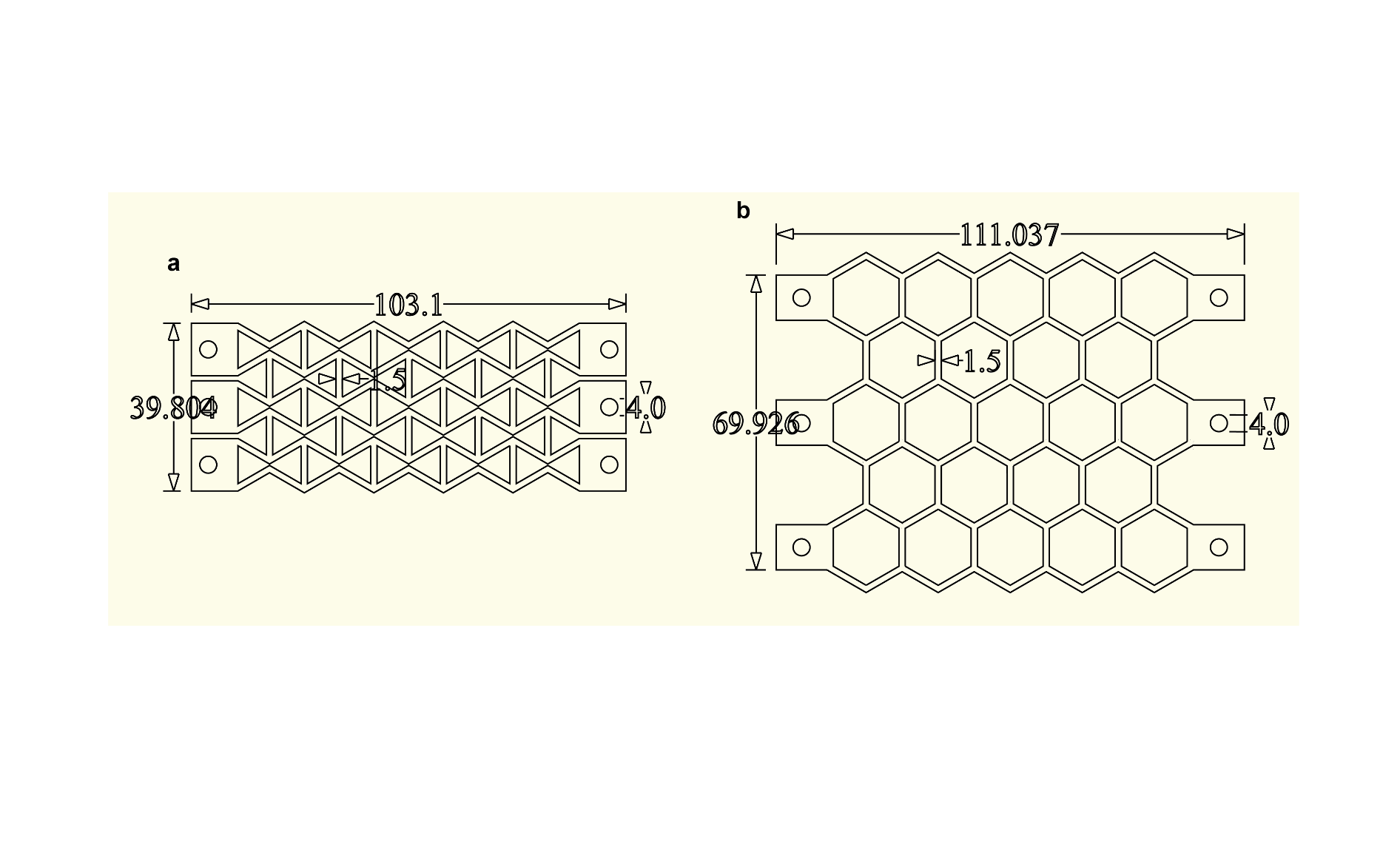


**Figure S1**. Dimensions in mm of a) the milled re-entrant auxetic and b) conventional honeycomb geometries with unit cell angles of 60° and 120°, respectively.

*Preparation of the CNT-coated PHU Composite Metamaterials:* To prepare the homogeneous dispersion for spray-coated metamaterials, the SWNTs are dispersed in ethanol at a concentration of 1 mg mL^-1^ using a SONOPULS ultrasonic homogenizer (Bandelin electronic GmbH & Co. KG, Berlin, Germany). A spray gun with a 1 mm nozzle size and 2.5 bar pressure is used to deposit the SWNTs solution onto the shape reconfigurable architecture.

*Photothermal Heating:* Photothermal effects are studied by measuring the surface temperature of the composite metamaterial under the irradiation of a 250 W infrared heat lamp with ø 35 cm aluminium reflector (Albert Kerbl GmbH, Germany) using a forward looking infrared camera (FLIR 655sc, FLIR Systems) and analyzed with the FLIR Research Studio analysis tool.

*Electrothermal Joule Heating:* The thin Cu-electrodes are glued by silver paste on both ends of the SWNT-coated metamaterials, and then connected into a closed circuit with a DC power supply (Basetech Bt-305). The thermal properties of the composite metamaterials are measured with a FLIR camera.

The relationship between the applied voltage and the temperature (*T*_s_) of the steady states is established using Equation S2, based on the energy balance of Joule heating versus power dissipation^[2,3]^:

$T_{s}=T_{0}+U^{2}/R\alpha A$ (S2)

where *U*, *T*_0_, *R*, *α*, and *A* are the supplied voltage, initial ambient temperature, resistance of the metamaterial, heat-transfer coefficient and heating area, respectively.

*Finite Element Simulation:* FE simulation of the individual displacement and heating is conducted using the structural mechanics module of COMSOL Multiphysics 6.0. First, the specimen is deformed by applying displacement on both sides. This displacement is then kept constant for 1 hour. Afterwards the samples are heated. For the global heating, constant heat flux is applied on all boundaries which corresponds to the oven heating. For the Joule heating, voltage is applied on the one side of the desired electrode positions while the other side is grounded. After short time, steady-state temperatures are obtained, which further translate into temperature-dependent stress relaxation. To this end, the experimentally determined activation energy and relaxation times are inputted. After 1 hour of applied voltage under deformation, the deformation is released until zero stress is reached. Afterwards, the reconfigured sample is stretched a second time to calculate the Poisson's ratio after reconfiguration. During the individual steps, temperature and stress are probed for each individual line. All input parameters are summarized in the Table S1.

**Table S1.** Input parameters for the FE simulations for the prediction of the stress relaxation of the CAN metamaterials during the localized Joule heating.

| **Property** | **Symbol** | **Value** | **Unit** |
| --- | --- | --- | --- |
| Bulk Poisson's Ratio | *ν* | 0.3 | - |
| Young's Modulus | *E* | 320 | kPa |
| Instantaneous Shear Modulus | *G*_inst_ | 123 | kPa |
| Bulk Modulus | *K* | 267 | kPa |
| Stretch Displacement | Δ*L*_x_ | 11.8 | mm |
| Activation Energy | *E*_a_ | 81 | kJ/mol |
| Relaxation Time | *τ** | 135 | h |
| Heat Flux | *q_0_* | 300 | W/m^2^ |
| Voltage | *V* | 32 | V |
| Emissivity | *ε* | 0.7 | - |

**2. Characterization Methods**

*Attenuated total reflection infrared spectroscopy (ATR)* was performed using a JASCO FT/IR-4100 spectrometer, with the scanning range of 600 – 4000 cm^-1^ and resolution of 4 cm^-1^ over 32 scans.

*Dynamic mechanical analyses (DMA)* is performed using a MCR 702 MultiDrive from Anton Paar with tensile loading. The **stress relaxation tests** at various temperature from 110 °C to 150 °C are measured on DMA, and dog-bone-shaped samples with dimensions of 10×4×2 mm^3^ (length × width × thickness) are pre-loaded by a 0.001 N force to ensure straightness. The samples are stretched to 4 %, and the strain is held constant during the test. Experiments on **creep recovery** are conducted by alternating the applied stress between 0 kPa and 2.8 kPa. In a standard test, a stress of 2.8 kPa is maintained for 120 seconds, followed by a 180-second recovery period This cycle is repeated multiple times, with the corresponding strain profile recorded over time. The storage modulus *E'* and loss factor tan *δ* are measured at a fixed frequency of 1 Hz while performing a **temperature ramp** from -80 °C to 130 °C with a heating rate of 3 °C min^-1^ in temperature to determine *T*_g_.

*Tensile mechanical properties* are measured with a Shimadzu compact tabletop testing machine EZ Test using a 5 N load cell and displacement rate of 10 mm s^-1^. For the **cyclic tensile test**, both loading and unloading processes are performed at a strain rate of 10 mm s^-1^. The cyclic tensile deformation treatment is executed in a stepwise manner, with progressively higher tensile strains applied until the targeted final strain is reached in the last cycle. Four dog-bone specimens are tested, and the standard error is calculated.

*Self-healing test:* Samples are cut from the middle using a sharp blade. Then, the fresh-cut surfaces are placed together at 25 °C followed by heating at 140 °C for 30 minutes. Healing efficiency (*η*) is quantitively calculated by the following equation:

$\eta=\frac{\varepsilon_{heal}}{\varepsilon_{pris}} \times100$ S3)

where is *ε*_heal_ the fracture strain for healed specimens, and *ε*_pris_ is the fracture strain for the pristine specimens.

*^1^H and ^13^C NMR* spectra are recorded on a Bruker Avance DPX spectrometer at 300 MHz. Chemical shifts are shown as relative to the residual non-deuterated species of the used solvent: dimethylsulfoxide-*d_6_* ((CD_3_)_2_SO: *δ*(^1^H) = 2.50 ppm), chloroform-*d* (CDCl_3_: *δ*(^1^H) = 7.26 ppm).

*Determination of the Poisson's Ratio:* The deformation of the shape morphing geometry is monitored by taking photographs (Panasonic DMC-G70KAEGK camera) at different elongations with local strains *ε*_xx_ and *ε*_yy_ measured within the inner-most unit of the geometry (e.g., Representative Volume Element (RVE), highlighted in yellow in Figure S3) to avoid boundary effects.^[4]^ The Poisson's ratio is calculated by the following equation: $\nu=\frac{\varepsilon_{yy}}{\varepsilon_{xx}}=-{\frac{v_{top}-v_{bottom}}{h}}/{\frac{u_{right}-u_{left}}{l}}$ (S4).


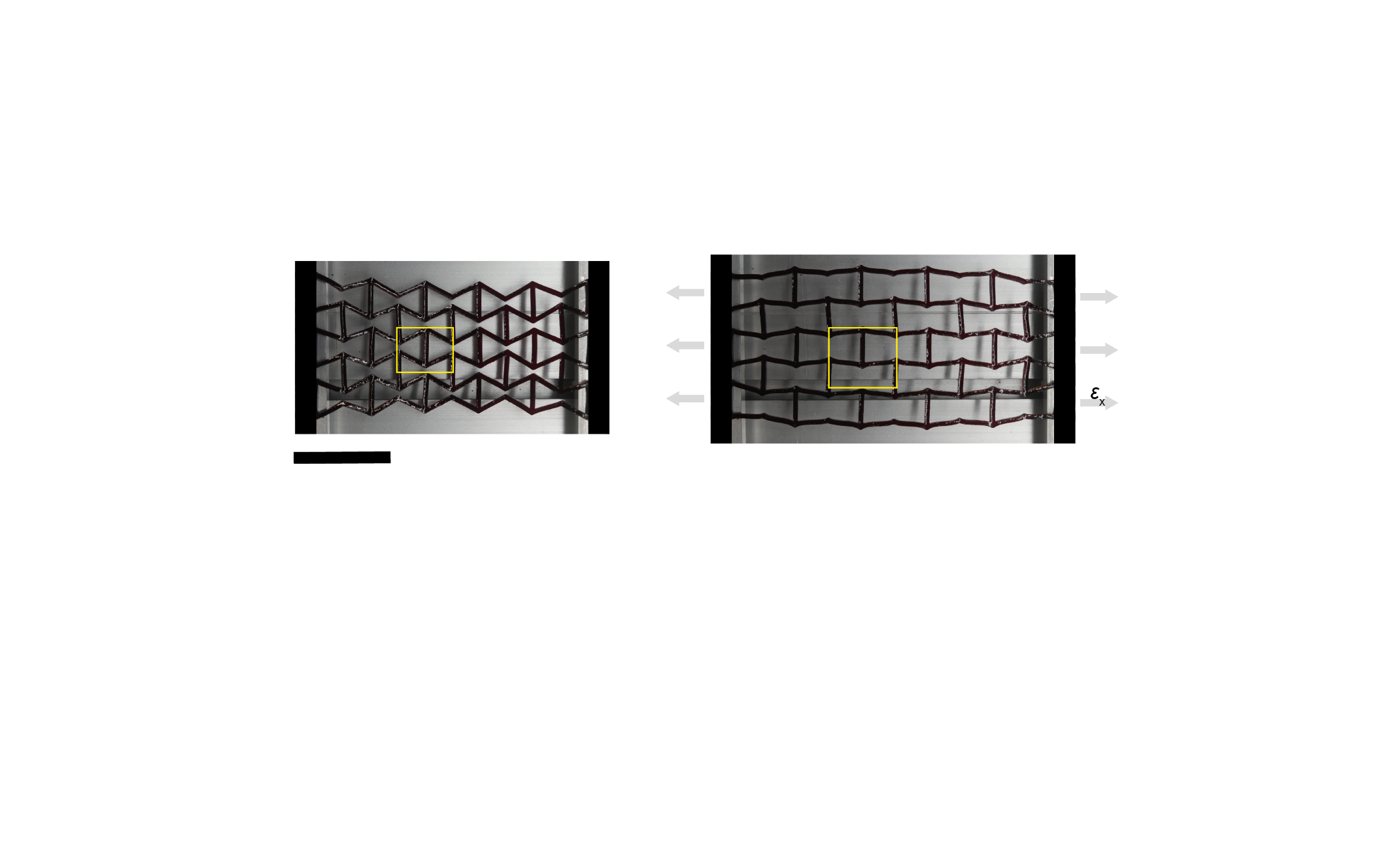


**Figure S2.** (left) The fabricated re-entrant auxetic structure in the unstretched state and the representative volume element (RVE) highlighted as yellow. (right) The deformed structure and the RVE under uniaxial stretching along the x-axis. (*ν* = -1.3 at *ε*_x_ = 28 %). The scale bar is 30 mm.

**3. Supplementary Analysis**


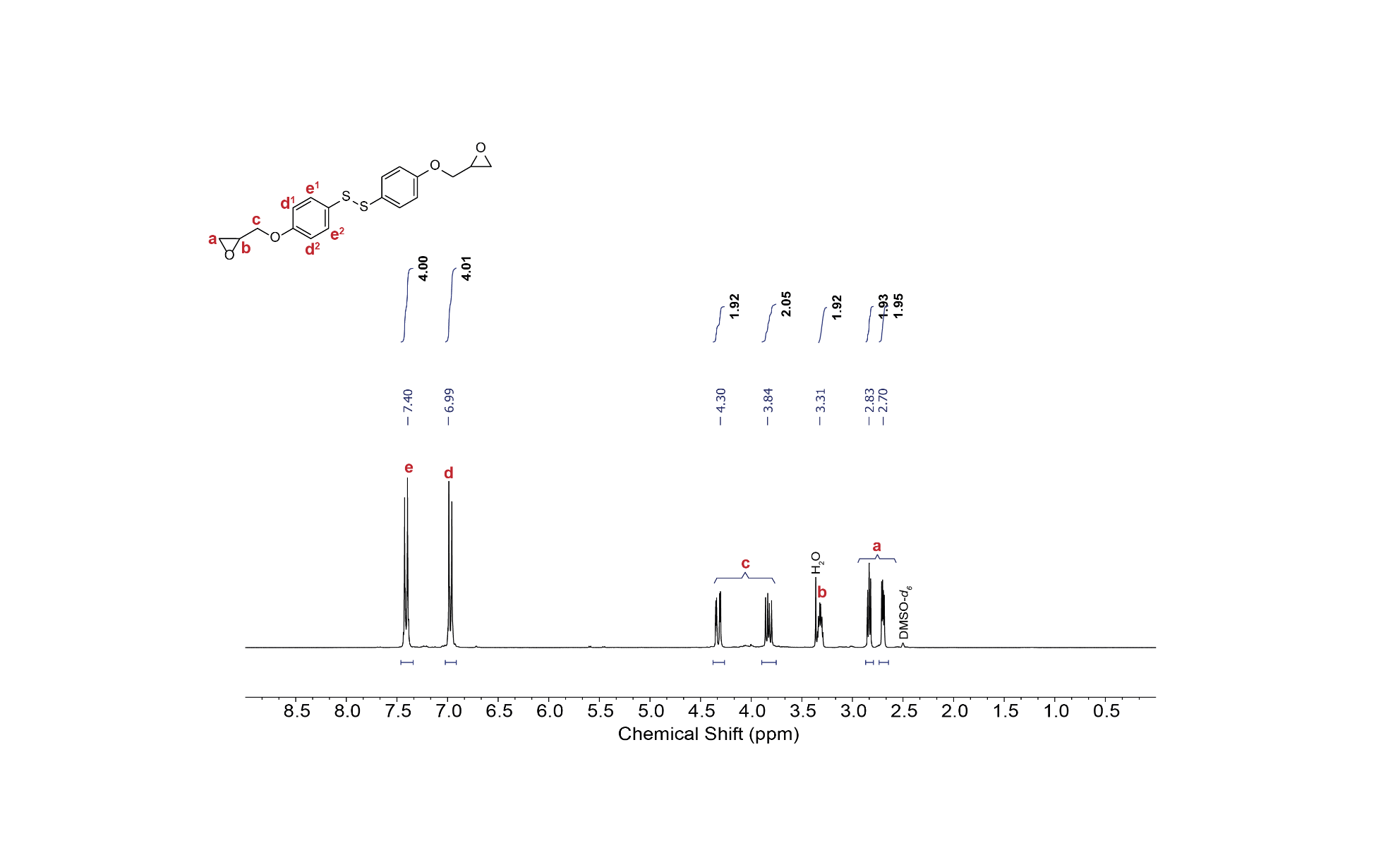


**Figure S3.** ^1^H NMR spectrum of the **precursor** in DMSO-*d_6_*.


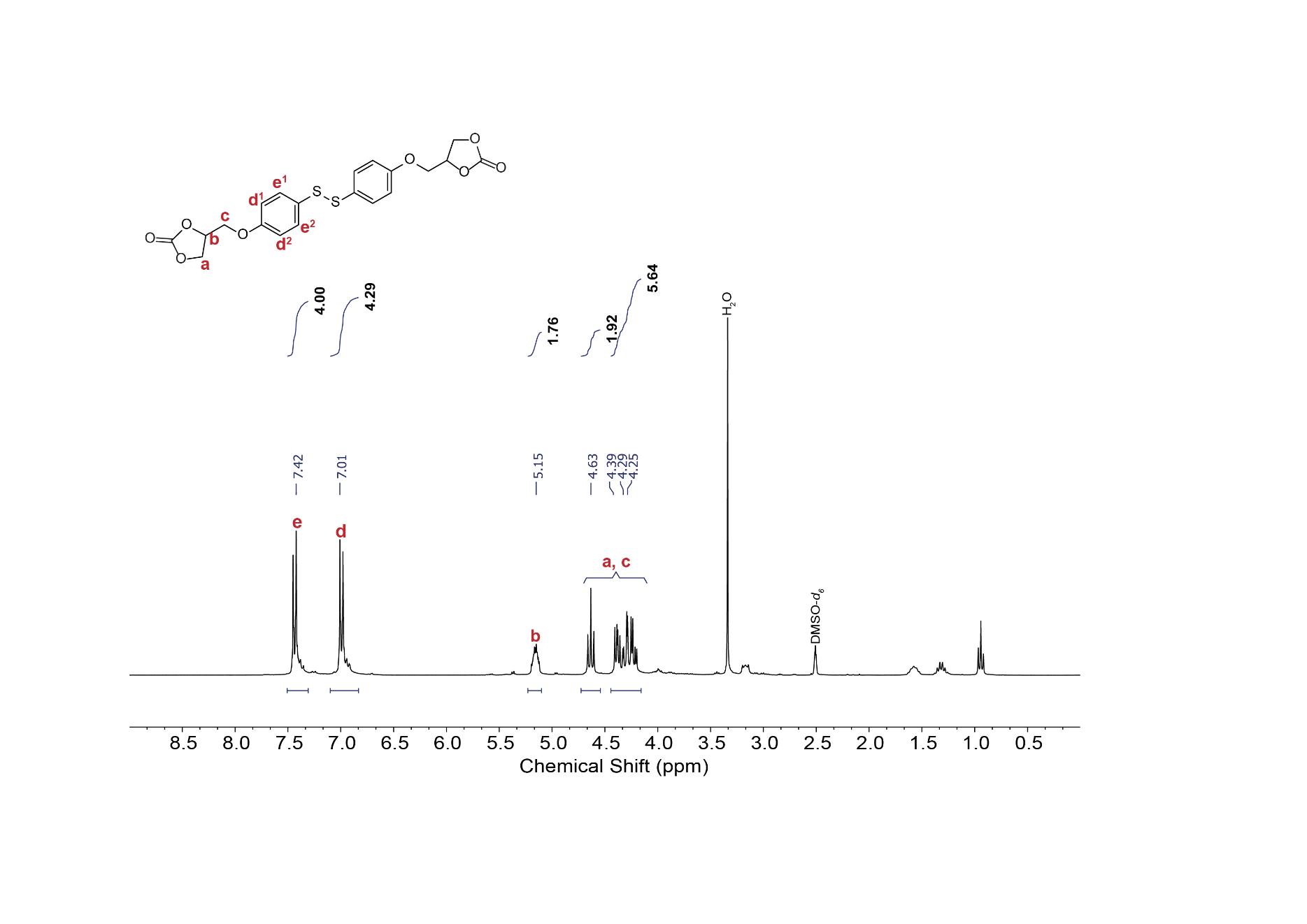


**Figure S4.** ^1^H NMR spectrum of **BisC_5_-SS** in DMSO-*d_6_*. Residual peaks at 0.95, 1.3, 1.56 and 3.15 ppm are assigned to the *n*Bu_4_NI catalyst.

*Further comments:* The crude monomer is intentionally used as *n*Bu_4_NI is known to ideally accelerate disulfide metathesis.


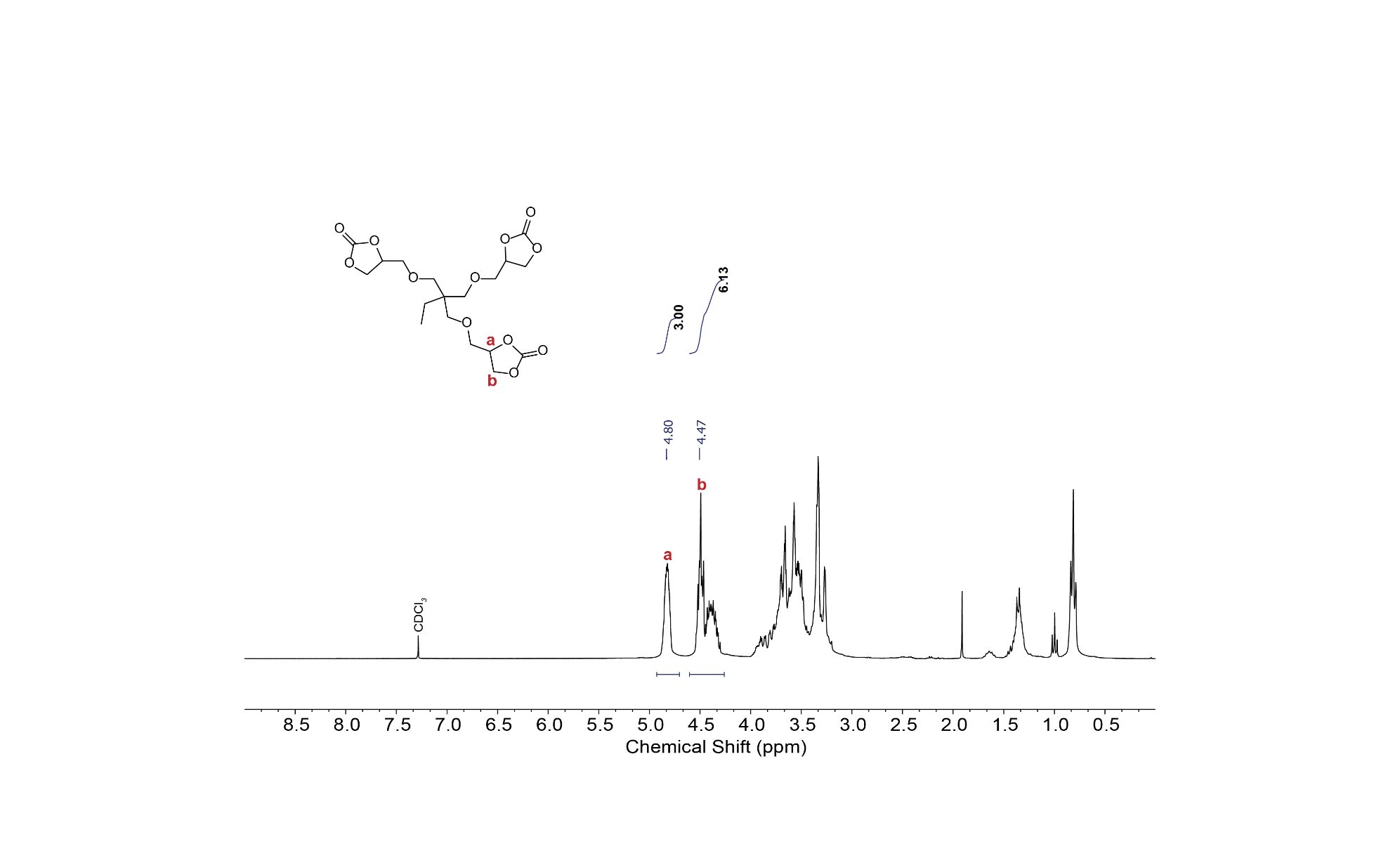


**Figure S5.** ^1^H NMR spectrum of **TrisC_5_** in CDCl_3_.


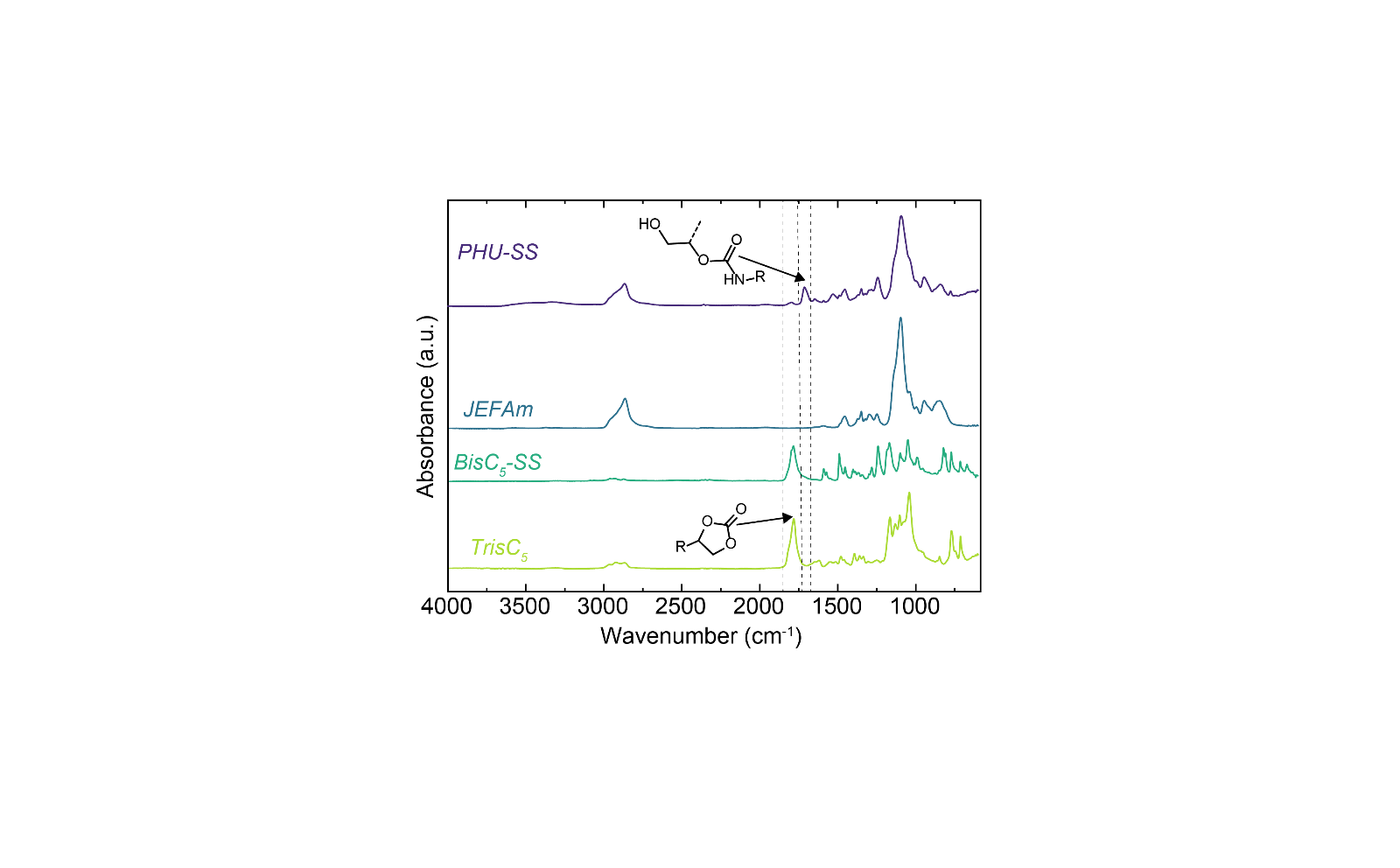


**Figure S6.** ATR-IR spectra of the precursors and cross-linked PHU elastomer.


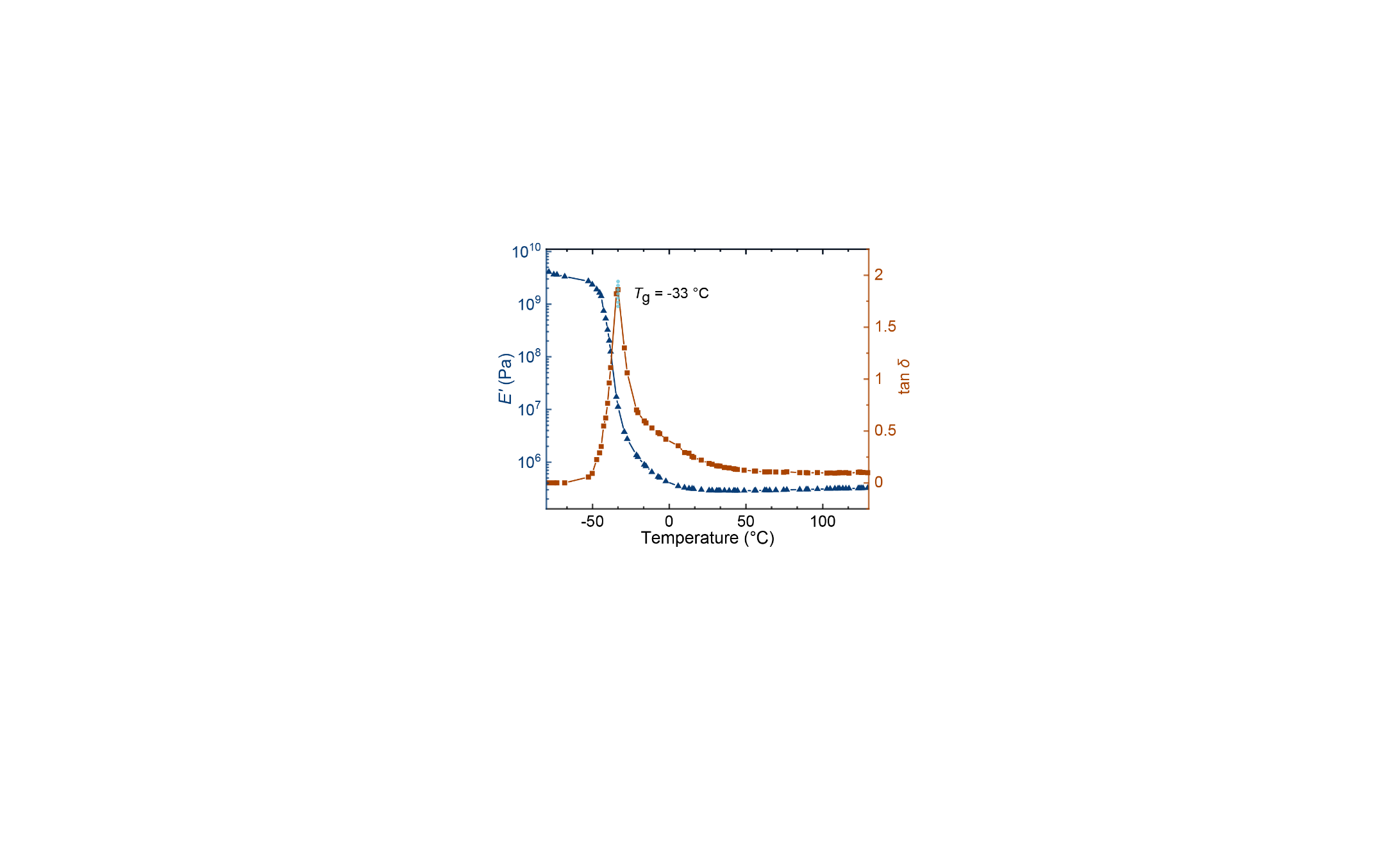


**Figure S7.** Temperature dependence of the storage modulus (*E′*) and loss factor (tan *δ*) for the dynamic PHU elastomer by dynamic mechanical analysis (DMA). The glass transition temperature (tan *δ* peak position) of *T*_g_ = – 33 °C shown in the graph.


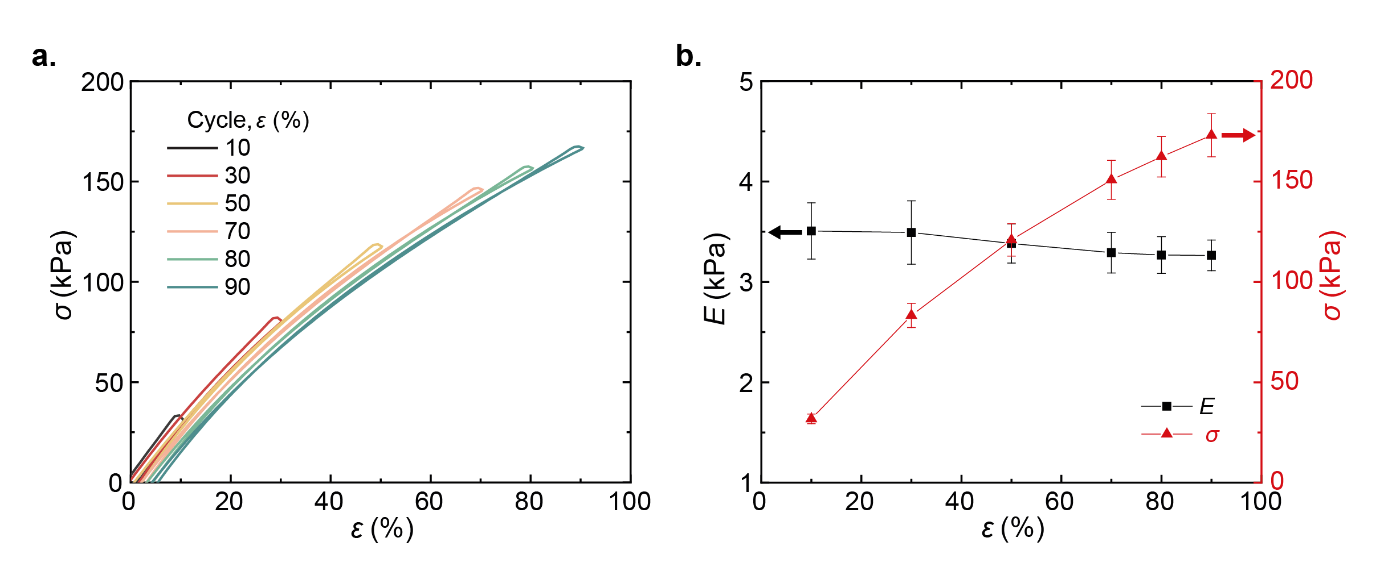


**Figure S8.** Mechanical properties of the PHU elastomer. a) Sequential cyclic stretching of the elastomer at different strains without an interval between each consecutive cycle and b) corresponding Young's modulus (*E*) and stress (*σ*) as a function of the applied strain for each loading cycle.


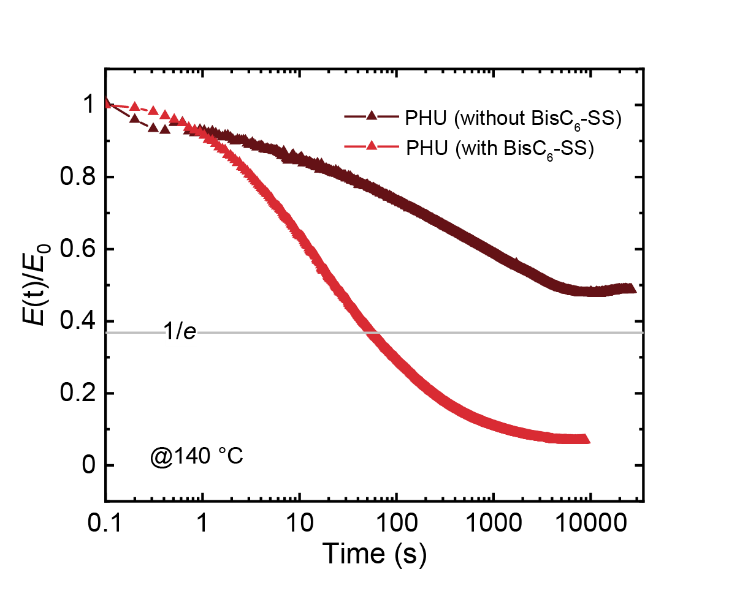


**Figure S9.** Normalized stress relaxation curves of the PHU (without BisC_6_-SS) and PHU (with BisC_6_-SS) elastomers at 140 °C, 1/*e* represents the stress relaxes to 1/*e* of the initial stress. Aromatic disulfide metathesis occurs, which allows the PHU elastomer consisting of BisC_6_-SS to relax stresses and flow.


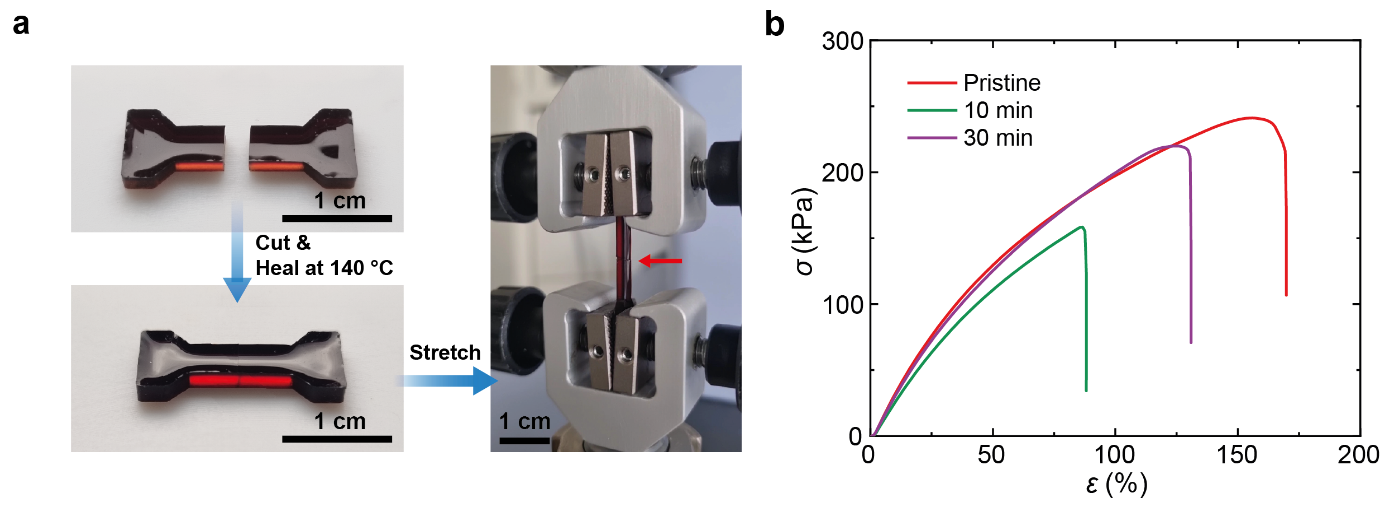


**Figure S10.** Self-healing properties of the PHU elastomer. a) Photographs of the self-healing process. The dogbone sample was cut into two pieces, and then the separated two parts were rejoined following heat treatment (140 °C for 30 min). The repaired PHU elastomer could be stretched over 120 %. The red arrow refers to the healing area. b) Tensile stress-strain curves of the pristine and healed (healing for 10 and 30 min at 140 °C) elastomer. As expected, a longer healing time resulted in a higher healing efficiency (from 55 % to 81 %).


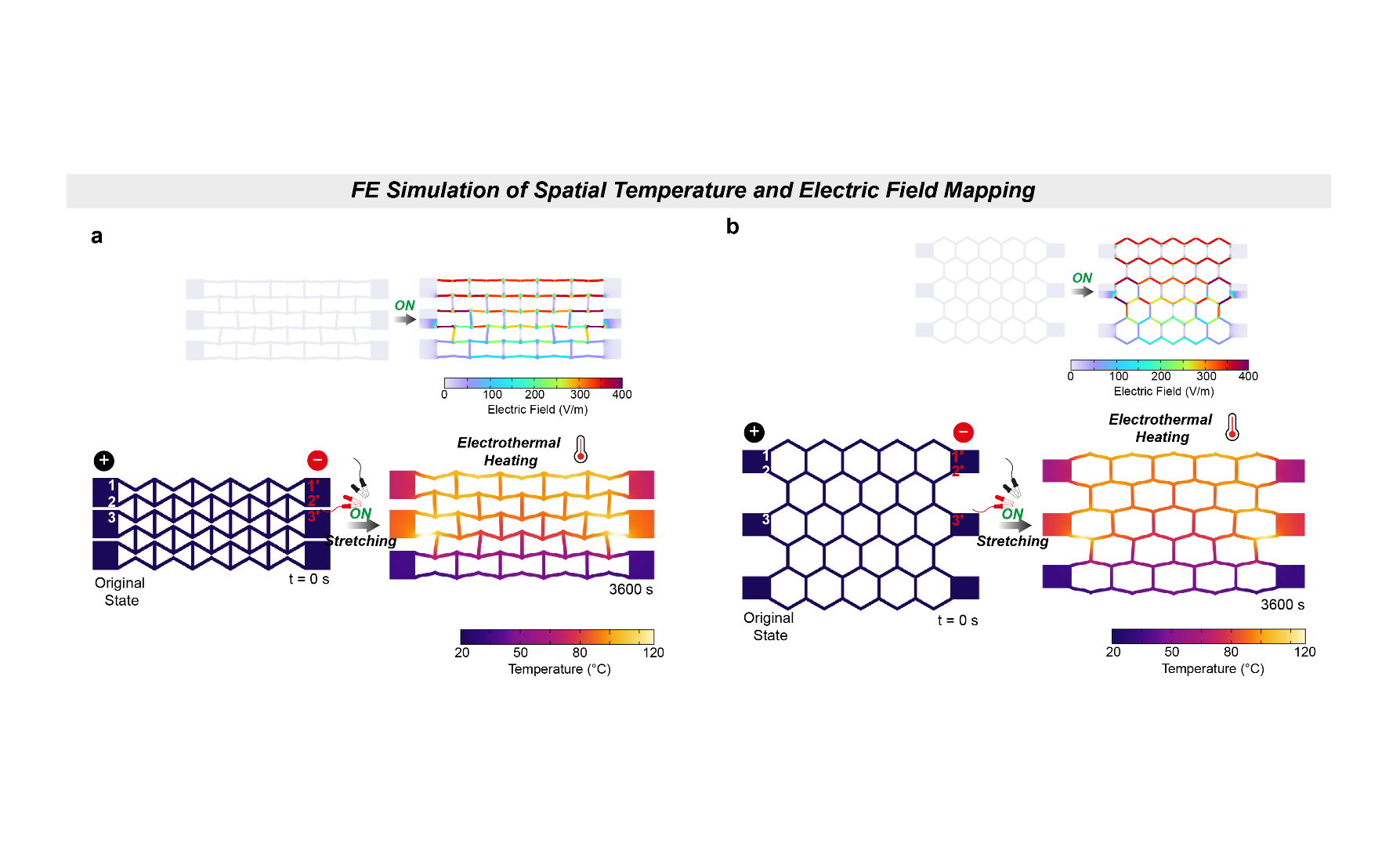
**Figure S11.** Electric field mapping on the reconfigurable a) re-entrant auxetic and b) honeycomb architectures during the electrothermal heating. The horizontal lines of the reconfigurable architectures that carry the electric current heat up to 100°C under the applied voltage of 32 V for 1 h.


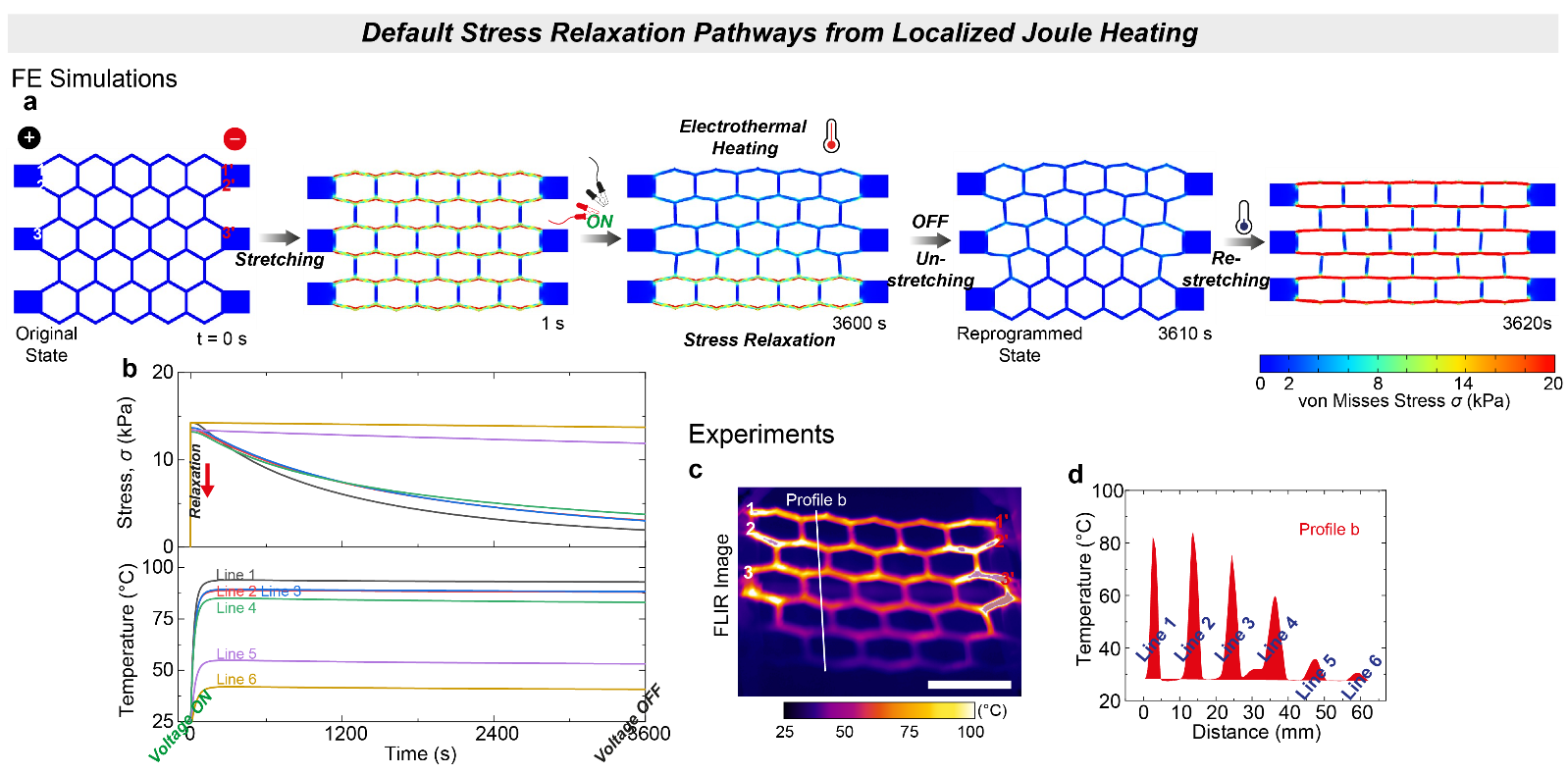


**Figure S12.** Electro-adaptive shape reconfiguration of the honeycomb composite metamaterial for creating permanent deformations only in spatially selected regions. a) FE simulations showcase the von Mises stress distribution of the honeycomb composite architecture during the electrothermal shape reprogramming: (i) placement of the electrodes, (ii) application of voltage under stretching, (iii) release of the applied force in the OFF state, and (iv) tensile reloading. b) Temperature evolution and the stress response on the re-entrant auxetic composite structure under an applied voltage of 32 V for 1 h obtained from FE analyses. The stress level across the entire structure uniformly increases upon restretching after a period of 3610 seconds. c) Infrared (IR) thermograph collected during Joule heating (within ca. 13 min) with the input voltage of 32 V at an applied strain of 10 %. The scale bar is 30 mm. d) Spatial temperature distribution of the honeycomb composite along the marked white line (profile b), extracted from (c).

**3 References**

[1] M. Bourguignon, B. Grignard, C. Detrembleur, *Angew. Chem. Int. Ed.* **2022**, *61*, e202213422.

[2] Y. Cheng, H. Zhang, R. Wang, X. Wang, H. Zhai, T. Wang, Q. Jin, J. Sun, *ACS Appl. Mater. Interfaces* **2016**, *8*, 32925–32933.

[3] D. Jiao, F. Lossada, J. Guo, O. Skarsetz, D. Hoenders, J. Liu, A. Walther, *Nat. Commun.* **2021**, *12*, 1312.

[4] D. Mousanezhad, S. Babaee, H. Ebrahimi, R. Ghosh, A. S. Hamouda, K. Bertoldi, A. Vaziri, *Sci Rep* **2015**, *5*, 18306.
